# Transcriptomics profile of human bronchial epithelial cells exposed to ambient fine particles and influenza virus (H3N2)

**DOI:** 10.1038/s41598-023-46724-6

**Published:** 2023-11-07

**Authors:** Yuan Liu, Yinbiao Wang, Rui Zhang, Shaolan Wang, Juan Li, Zhen An, Jie Song, Weidong Wu

**Affiliations:** School of Public Health, Xinxiang Medical University, Xinxiang, 453003 Henan Province China

**Keywords:** Microbiology, Environmental sciences, Molecular medicine

## Abstract

Fine particulate matter (PM_2.5_) pollution remains a major threat to public health. As the physical barrier against inhaled air pollutants, airway epithelium is a primary target for PM_2.5_ and influenza viruses, two major environmental insults. Recent studies have shown that PM_2.5_ and influenza viruses may interact to aggravate airway inflammation, an essential event in the pathogenesis of diverse pulmonary diseases. Airway epithelium plays a critical role in lung health and disorders. Thus far, the mechanisms for the interactive effect of PM_2.5_ and the influenza virus on gene transcription of airway epithelial cells have not been fully uncovered. In this present pilot study, the transcriptome sequencing approach was introduced to identify responsive genes following individual and co-exposure to PM_2.5_ and influenza A (H3N2) viruses in a human bronchial epithelial cell line (BEAS-2B). Enrichment analysis revealed the function of differentially expressed genes (DEGs). Specifically, the DEGs enriched in the xenobiotic metabolism by the cytochrome P450 pathway were linked to PM_2.5_ exposure. In contrast, the DEGs enriched in environmental information processing and human diseases, such as viral protein interaction with cytokines and cytokine receptors and epithelial cell signaling in bacterial infection, were significantly related to H3N2 exposure. Meanwhile, co-exposure to PM_2.5_ and H3N2 affected G protein-coupled receptors on the cell surface. Thus, the results from this study provides insights into PM_2.5_- and influenza virus-induced airway inflammation and potential mechanisms.

## Introduction

Particulate matter (PM) in ambient air with an aerodynamic diameter of less than or equal to 2.5 μm is referred to as PM_2.5_. Due to its small size and high specific surface area, PM_2.5_ can absorb diverse harmful substances from the air, including microbes, transition metals, and polycyclic aromatic hydrocarbons (PAH), then enters the lungs and other remote regions through the circulatory system^1–3^. Even though air pollution mitigation measures have achieved impressive gains in China in the last several years, PM_2.5_ remains a significant threat to human health^4^. A recent study has demonstrated that PM_2.5_ pollution still causes around 4.23 million deaths globally each year^5^. Previous study has shown that PM_2.5_ accounts for 96% of PM seen in human lung parenchyma^6^, directly causing or aggravating respiratory diseases. Long-term PM_2.5_ exposure has been associated with higher hospitalization and mortality for pneumonia, lung cancer, cardiovascular disorders, and neurological diseases^7–10^. A case-crossover study of 40,002 people in Guangzhou found that each 10 μg/m^3^ increase in PM_2.5_ concentration was related to a 1.6% increase in chronic obstructive pulmonary disease (COPD) hospitalization^11^. The interaction of PM_2.5_ and the influenza virus has been reported in multiple epidemiological investigations, showing a correlation between exposure to PM_2.5_ and higher hospitalization and mortality rates by respiratory viral infections^12–14^.

Influenza is a devastating respiratory viral disease that infects around one billion people each year and kills 500,000^15^. The most prevalent kind of influenza virus is influenza A virus (IAV), with H1N1 and H3N2 as the main subtypes causing influenza, pneumonia, and acute respiratory distress syndrome^16^. Incubation time for influenza viruses ranges from one to three days following infection of the host, and the development of influenza symptoms is largely dependent on the body's innate immune system's capacity to eliminate the virus^17^. Previous study has demonstrated that air pollution influences viral infection through modification of the viral life cycle or the intensity of the host's innate and adaptive immune response after infection^18^. Epidemiological investigations and experimental studies have demonstrated that exposure to environmental PM can result in the exacerbation of adverse health effects linked with respiratory viral infection in humans, and that metals in PM can interact with respiratory viruses via complicated modes of action leading to serious harm in humans^19,20^. For example, ozone (O_3_) exposure promotes the production of protein hydrolases, which more efficiently activate influenza virus particles, resulting in greater IAV infection^21^. PM_10_ increases H5N1 influenza virus infection in A549 cells (a human alveolar epithelial cell line) by modulating the innate immune response^22^. PM_2.5_ poses a negative impact on the innate immune system of the lung by altering the function of bronchial epithelial cells' mucus cilia, hindering alveolar macrophages' ability to destroy pathogens, reducing the natural killer (NK) cell response, and causing airway epithelial cell dysfunction^23–25^. By inhibiting lipopolysaccharide (LPS)-induced activation of the NLRP3 (NOD-, LRR- and pyrin domain-containing protein 3) inflammasome and production of interferon-β (IFN-β) during influenza infection, PM_2.5_ could also change the inflammatory responses of macrophages^26^. Taken together, these studies suggest that air pollutants can increase influenza virus infectivity and disease severity by inducing inflammation, suppressing host innate and adaptive immunity, reducing antiviral ability, and increasing virus replication.

Airway epithelial cells, a physical barrier against inhaled air pollutants, are the primary targets of environmental hazards including PM_2.5_ and influenza viruses^27^. In this study, we used a normal human bronchial epithelial cell line (BEAS-2B) as an in vitro model and transcriptomic approach to examine the joint effects of PM_2.5_ and H3N2 on the transcriptome of human airway epithelium to provide insights into the mechanisms for interactions of ambient PM_2.5_ and H3N2.

## Results

### Metallic elements of PM_2.5_

**Figure 1:**
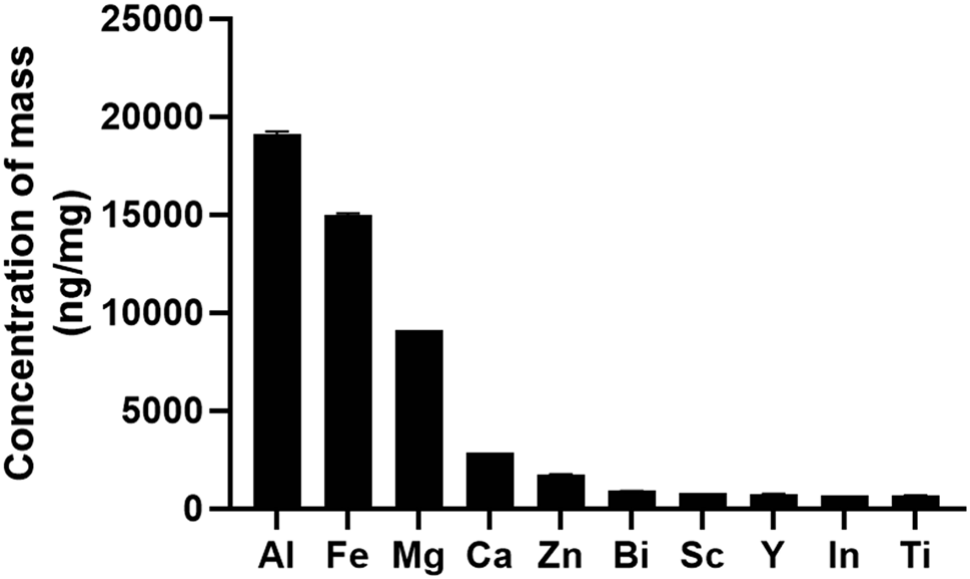
The top 10 Metallic elements composition of PM_2.5_ (${\overline{\text{X}}}$ ± SD, n = 3).

ICP-MS was used to detect the content of various metallic elements in PM_2.5_. As shown in Fig. 1, The top 10 metallic elements in terms of concentration in the PM_2.5_ samples utilized in this study were Al, Fe, Mg, Ca, Cu, Zn, Bi, Sc, Y, In, Ti. Of them, Al, Fe, Ca, and Mg are crustal elements, implying that road and construction specks of dust may have the greatest impact on air quality in the winter and spring seasons of Xinxiang, where the study was performed.

### Cytotoxicity of PM_2.5_ exposure and H3N2 infection on BEAS-2B cells

**Figure 2:**
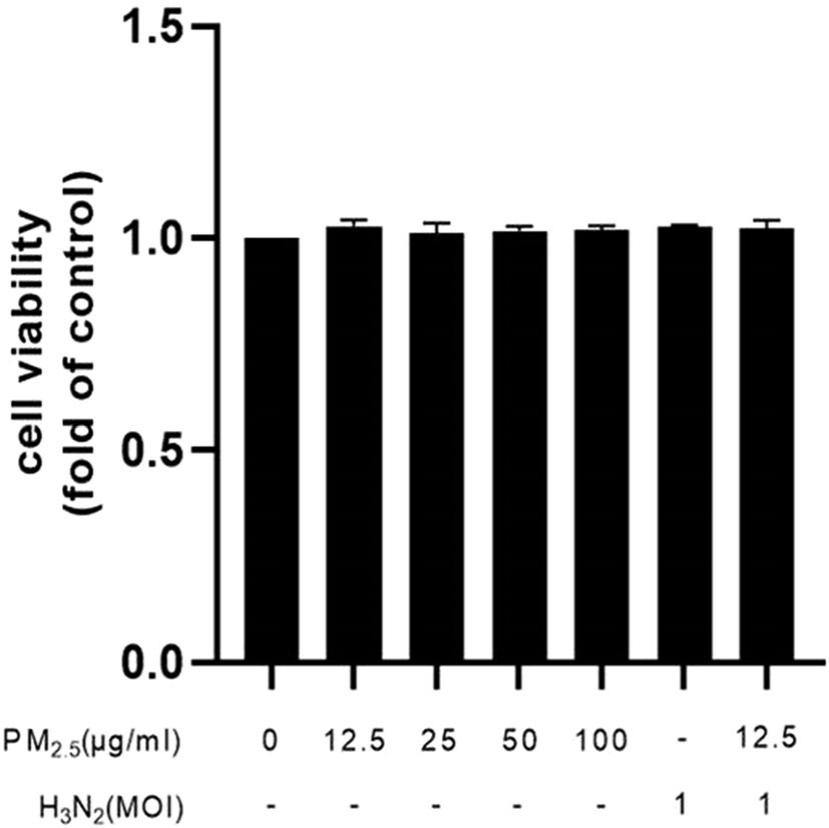
PM_2.5_ and H3N2 virus exposure caused damage in BEAS-2B cells: Cell viability (${\overline{\text{X}}}$ ± SD, n = 3).

CCK-8 assay was used to evaluate the cytotoxicity of PM_2.5_ exposure and H3N2 virus infection on BEAS-2B cells, which showed that exposure to PM_2.5_ (0–100 μg/mL) and H3N2 virus (MOI = 1) had minimal effect on cell viability (Fig. 2).

### Influence of PM_2.5_ on infectivity of H3N2 virus on BEAS-2B cells

**Figure 3:**
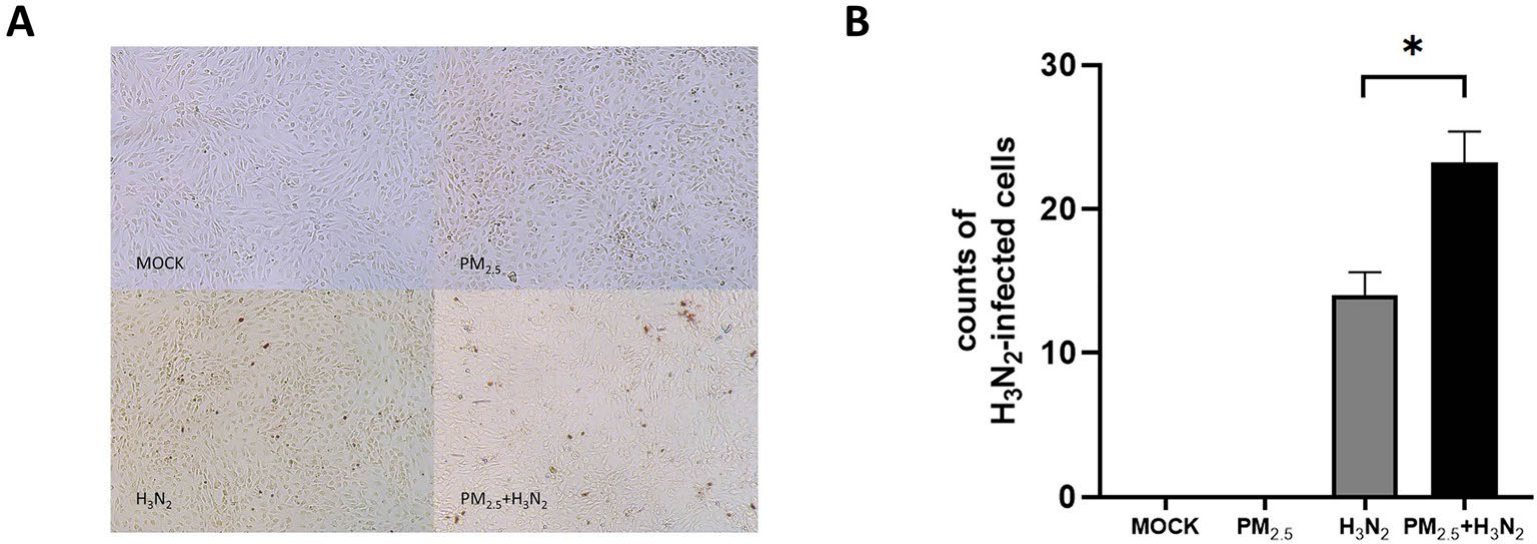
Influence of PM_2.5_ on infectivity of H3N2 virus BEAS-2B cells. (**A**) Cell morphology after IPMA. (**B**) PM_2.5_ exposure enhances H3N2 infectivity in BEAS-2B cells (n = 3, *P < 0.05, compared to H3N2 virus exposure).

To assess whether exposure to PM_2.5_ could affect the ability of the H3N2 virus to infiltrate BEAS-2B, we first exposed them to PM_2.5_ (12.5 g/mL) for 4 h before infecting them with H3N2 (MOI = 1). As shown in Fig. 3A and B, co-exposure of BEAS-2B cells to PM_2.5_ and H3N2 virus increased 65.9% of virus-infected cells compared to H3N2 group alone (*P* < 0.05). It suggests that exposure to PM_2.5_ can increase the susceptibility of respiratory epithelial cells to IAV in co-exposure. This is consistent with what we found in our previous study^28^.

### Analyses of gene expression and correlations

**Figure 4:**
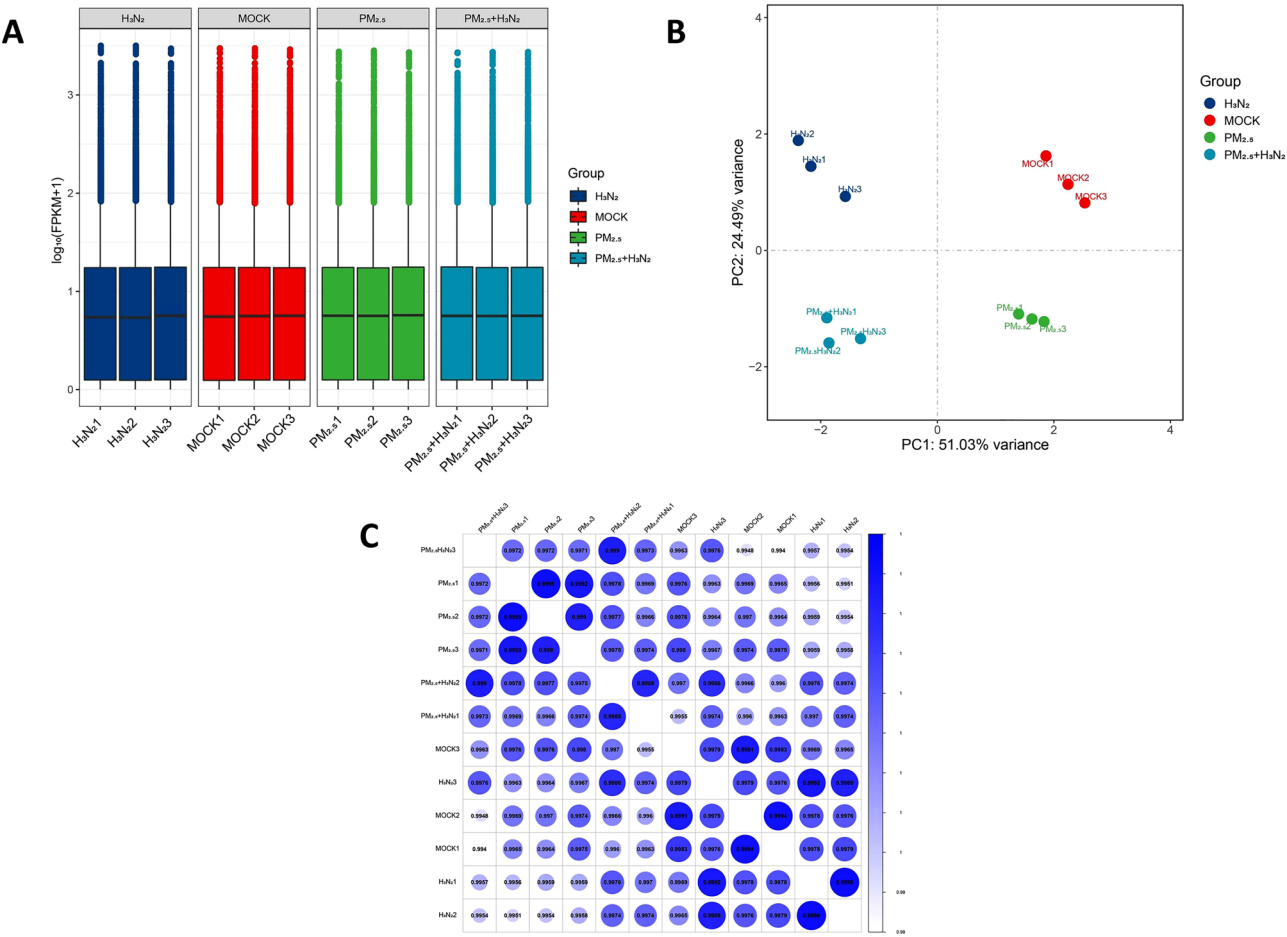
Gene expression level, principal component analysis, and correlations among samples. (**A**) The distribution of expression data from all the samples. (**B**) Principal component analysis (PCA). (**C**) The correlations among samples were analyzed by Pearson correlation.

The distribution of the gene expression data for all samples is displayed in boxplots. Gene expression levels of all samples were essentially the same after normalization, indicating that batch effect and systematic bias were not significant (Fig. 4A). The results of the principal component analysis (PCA) revealed that the control (MOCK) group, H3N2 group, PM_2.5_ group, and the co-exposure group (PM_2.5_ + H3N2) were well separated, indicating that there was a high degree of similarity among the samples in the same group and differences among different groups (Fig. 4B). As shown in Fig. 4C, the correlation coefficient between the samples was calculated by Pearson correlation analysis, and the closer the correlation coefficient is to 1, the higher the similarity between the samples, and the smaller the differences between the samples. The results show that biological experimental operations of the samples are highly repeatable and that samples in the same experimental group have a high degree of similarity.

### Screen of DEGs

**Figure 5:**
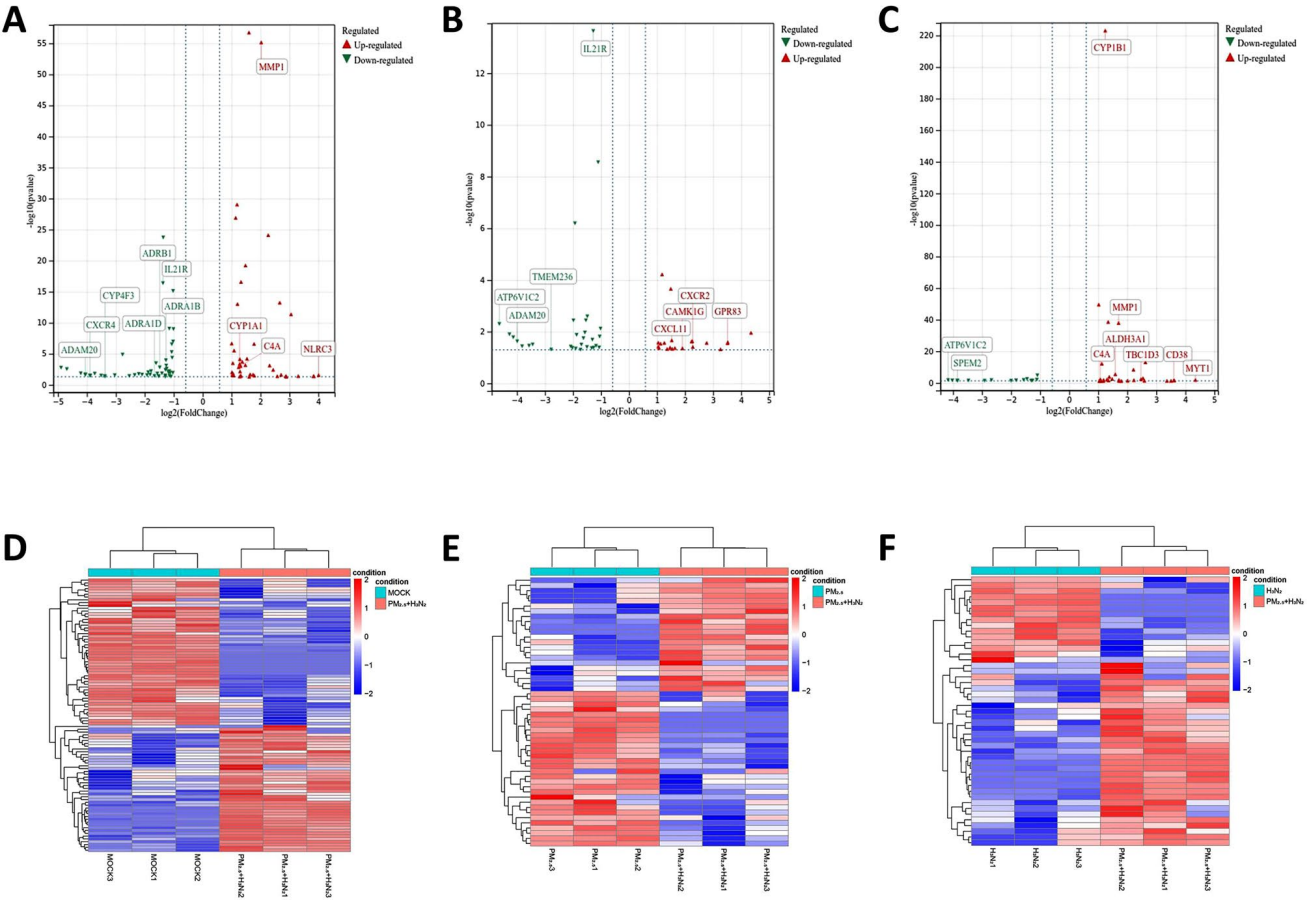
Volcanic map of DEGs expression profiles among (**A**) Co-exposure and MOCK. (**B**) Co-exposure and PM_2.5_ exposure. (**C**) Co-exposure and H3N2 virus exposure. And hierarchical cluster analysis heat map of DEGs expression profiles among (**D**) Co-exposure and MOCK. (**E**) Co-exposure and PM_2.5_ exposure. (**F**) Co-exposure and H3N2 virus exposure. (*P* < 0.05, |log_2_FC|> 1).

In PM_2.5_ group, 53 DEGs were detected in contrast to the control group, of which 30 were up-regulated and 23 were down-regulated. In H3N2 group, 54 DEGs were detected with 21 up-regulated and 33 down-regulated. In the co-exposure group, 97 DEGs were discovered compared with the control group, of which 45 were up-regulated and 52 down-regulated. Moreover, 52 DEGs were found between the co-exposure group and PM_2.5_ group, of which 22 were up-regulated and 30 down-regulated. 47 DEGs were found between the co-exposure group and H3N2 groups, of which 32 were up-regulated and 15 down-regulated (Fig. 5A–C). These DEGs could be divided into multiple sub-groups through a heat map of hierarchical cluster analysis (Fig. 5D–F). The top 10 DEGs in each comparison group are displayed in Tables 1, 2, 3.

**Table 1:** Top 10 DEGs between co-exposure group and MOCK group.

Gene ID	Log_2_ fold change	*P*-value	Regulation	Description
*MYT1*	4.303932	0.01205	Up	Myelin transcription factor 1
*NLRC3*	4.008194	0.026464	Up	NLR family CARD domain containing 3
*GCM1*	− 3.88936	0.033104	Down	Glial cells missing transcription factor 1
*CXCR4*	− 3.89126	0.033771	Down	C-X-C motif chemokine receptor 4
*FAM156B*	− 4.05245	0.022163	Down	Family with sequence similarity 156 member B
*ANKRD34B*	− 4.05414	0.022075	Down	Ankyrin repeat domain 34B
*ADAM20*	− 4.05763	0.022873	Down	ADAM metallopeptidase domain 20
*SPAG17*	− 4.21283	0.014399	Down	Sperm associated antigen 17
*ATP6V1C2*	− 4.69288	0.002946	Down	ATPase H + transporting V1 subunit C2
*SLC9A4*	− 4.89332	0.001712	Down	Solute carrier family 9 member A4

**Table 2:** Top 10 DEGs between co-exposure group and PM_2.5_ exposure group.

Gene ID	Log_2_ fold change	*P*-value	Regulation	Description
*TBC1D3H*	4.894203	0.002049	Up	TBC1 domain family member 3H
*ZBP1*	4.351898	0.010975	Up	Z-DNA binding protein 1
*STEAP4*	3.523135	0.025576	Up	STEAP4 metalloreductase
*GPR83*	3.514048	0.027748	Up	G protein-coupled receptor 83
*SOWAHB*	− 3.56796	0.03485	Down	Sosondowah ankyrin repeat domain family member B
*PTTG2*	− 3.81632	0.036827	Down	Pituitary tumor-transforming 2
*ADAM20*	− 3.99146	0.023591	Down	ADAM metallopeptidase domain 20
*B3GAT2*	− 4.13201	0.016485	Down	Beta-1,3-glucuronyltransferase 2
*QPRT*	− 4.26867	0.012643	Down	Quinolinate phosphoribosyltransferase
*ATP6V1C2*	− 4.62835	0.005014	Down	ATPase H^+^ transporting V1 subunit C2

**Table 3:** Top 10 DEGs between co-exposure group and H3N2 exposure group.

Gene ID	Log_2_ fold change	*P*-value	Regulation	Description
*TBC1D3H*	4.865379	0.002583	Up	TBC1 domain family member 3H
*MYT1*	4.344997	0.01179	Up	Myelin transcription factor 1
*KCNK13*	3.616799	0.023439	Up	Potassium two pore domain channel subfamily K member 13
*CD38*	3.59755	0.026918	Up	CD38 molecule
*LUM*	3.503359	0.041209	Up	Lumican
*HCRTR1*	− 3.46801	0.032	Down	Hypocretin receptor 1
*ANKRD34B*	− 3.84559	0.037877	Down	Ankyrin repeat domain 34B
*SPEM2*	− 3.84969	0.040319	Down	SPEM family member 2
*MTNR1A*	− 4.02484	0.02681	Down	Melatonin receptor 1A
*ATP6V1C2*	− 4.16972	0.017146	Down	ATPase H^+^ transporting V1 subunit C2

### GO analysis of DEGs

**Figure 6:**
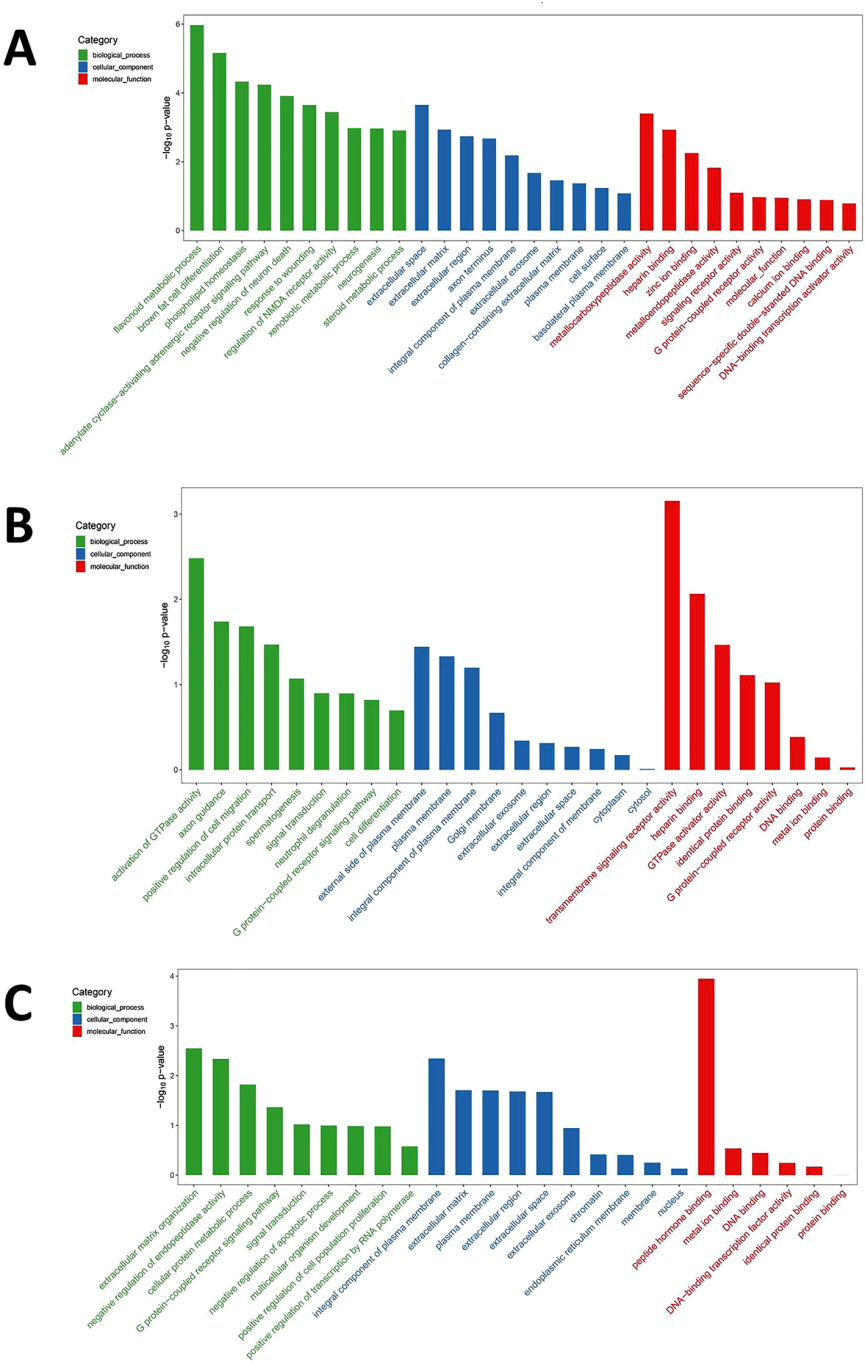
GO enrichment analysis of DEGs between (**A**) Co-exposure and MOCK. (**B**) Co-exposure and PM_2.5_ exposure. (**C**) Co-exposure and H3N2 virus exposure (Top 30 GO term).

The DEGs were further analyzed by GO enrichment analysis for their expression and functions. Hypergeometric test between the co-exposure group and the control group revealed 251 significant enrichment sub-classes related to the biological process (73.71%), molecular function (17.53%), and cellular components (7.37%), such as extracellular matrix organization, cellular protein metabolism, G protein-coupled receptor signaling pathway, and peptide hormone binding (Fig. 6A).

Between the co-exposure group and PM_2.5_ group, 103 enrichment sub-classes were identified, which were related to the biological process (59.22%), molecular function (25.24%), and cellular component (15.54%), such as transmembrane signaling receptor activity, activation of GTPase activity, positive regulation of cell migration, intracellular protein transport, and plasma membrane (Fig. 6B).

Between the co-exposure group and H3N2 group, 119 enrichment sub-classes were identified, which were associated with the biological process accounting (68.07%), cellular component (8.10%), and molecular function (23.53%), such as extracellular matrix organization, cellular protein metabolism, G protein-coupled receptor signaling pathway, and peptide hormone binding (Fig. 6C).

### KEGG pathway analysis of DEGs

**Figure 7:**
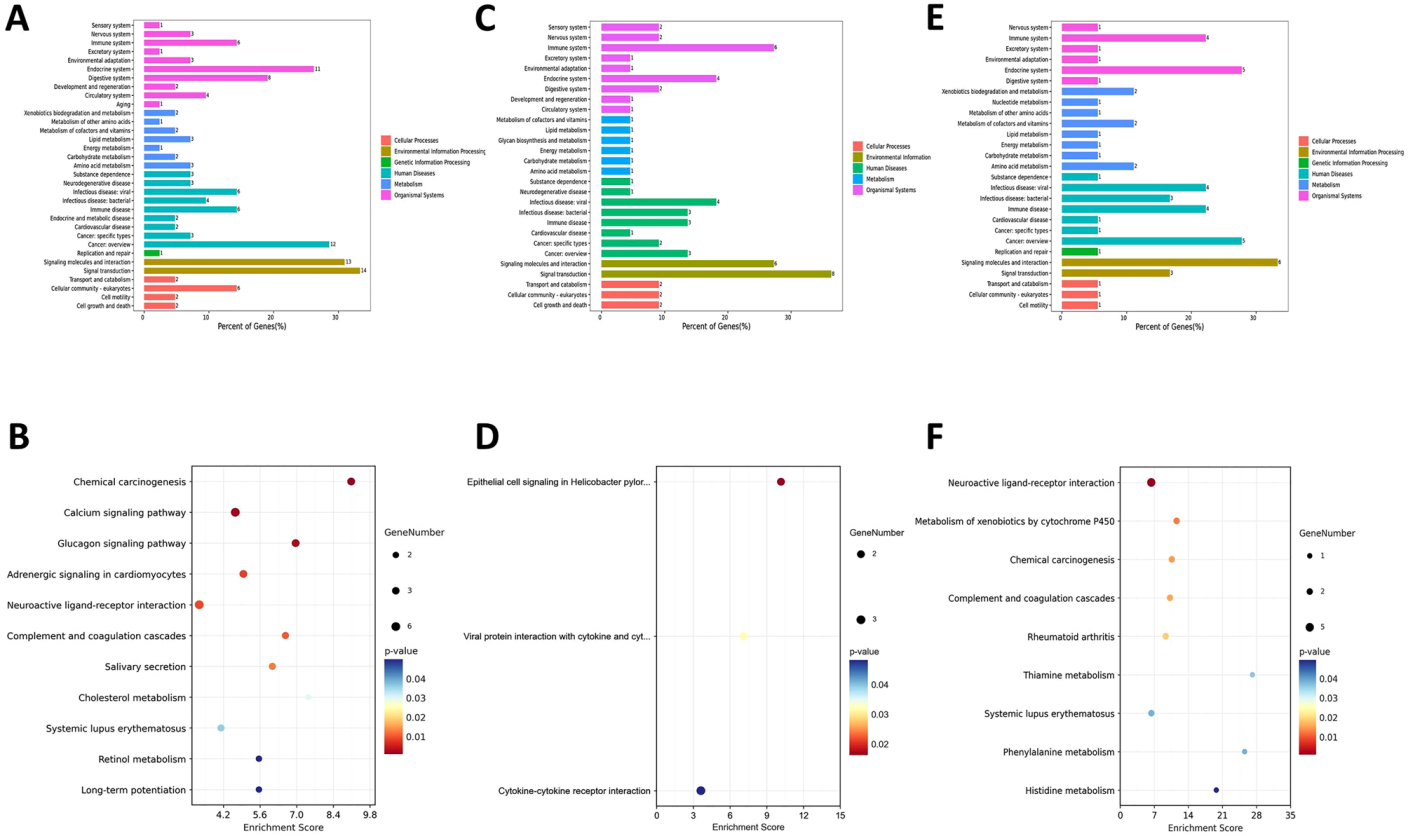
KEGG pathway classification (upper panel) and enrichment analysis (lower panel) of DEGs between Co-exposure and MOCK (**A** and **B**), Co-exposure and PM_2.5_ exposure (**C** and **D**), and Co-exposure and H3N2 virus exposure (**E** and **F**).

Analysis of DEG-associated pathways was performed using the KEGG database to study the potential mechanism of the joint biological effects of PM_2.5_ exposure and H3N2 infection. It was revealed that biological systems related to DEGs between the co-exposure and the control groups included the endocrine system, digestive system, and immune system. Related metabolic processes including lipid metabolism, carbon metabolism, etc. DEG-related human diseases include viral, bacterial, and immune diseases. DEG-related environmental information processing included signal transduction, signal molecules and interaction, and cellular community-eukaryotes of cellular processes (Fig. 7A). Moreover, DEGs were significantly enriched in several related pathways (levle3), including the calcium signaling pathway, neuroactive ligand-receptor interaction, complement and coagulation cascades, long-term potentiation, glucagon signaling pathway, and chemical carcinogenesis, among others. (Fig. 7B).

The biological systems associated with DEGs between the co-exposure and PM_2.5_ groups included the immune system and endocrine system. DEG-associated human diseases included viral, bacterial, immune, and cancer. DEG-associated environmental information processing included signal transduction, signal molecules and interaction (Fig. 7C). Moreover, DEGs were significantly enriched in several related pathways (levle3), including the cytokine-cytokine receptor interaction, viral protein interaction with cytokine and cytokine receptors and epithelial cell signaling in Helicobacter pylori infection (Fig. 7D).

Biological systems associated with DEGs between the co-exposure and H3N2 groups included the immune system and the endocrine system. DEG-associated human diseases included viral, bacterial, immune, and cancer. In addition, DEG-associated environmental information processing included signal transduction, and signal molecules and interaction (Fig. 7E). DEGs were significantly enriched in several related pathways (levle3), including neuroactive ligand-receptor interaction, metabolism of xenobiotics by cytochrome P450, complement and coagulation cascades, chemical carcinogenesis, rheumatoid arthritis, and systemic lupus erythematosus, et al. (Fig. 7F).

It is noteworthy that some differential genes and pathways were identified and enriched only in the co-exposure group through the discovery of DEGs and KEGG enrichment, KEGG pathways such as calcium signaling pathway, long-term potentiation, etc., and related DEGs including *Adrenoceptor Alpha 1 Beta* (*ADRA1B*)*, Adrenoceptor Beta 1* (*ADRB1*)*, Calcium/Calmodulin Dependent Protein Kinase II Beta* (*CAMK2B*), etc., implying that the co-exposure of PM_2.5_ and H3N2 is more than just an additive effect.

### Gene set enrichment analysis (GESA) of DEGs

The samples were split into two groups in the gene expression matrix that served as the input for the GSEA analysis. Then, the genes in these two groups were all listed based on their values generated from GSEA analysis from large to small, respectively. Here, we can tell whether a pathway is activated or inhibited by simply looking at the value of its corresponding gene in the list. The gene with a large value was putatively considered as up-regulated. On the contrary, the gene with a smaller value was considered down-regulated.

**Figure 8:**
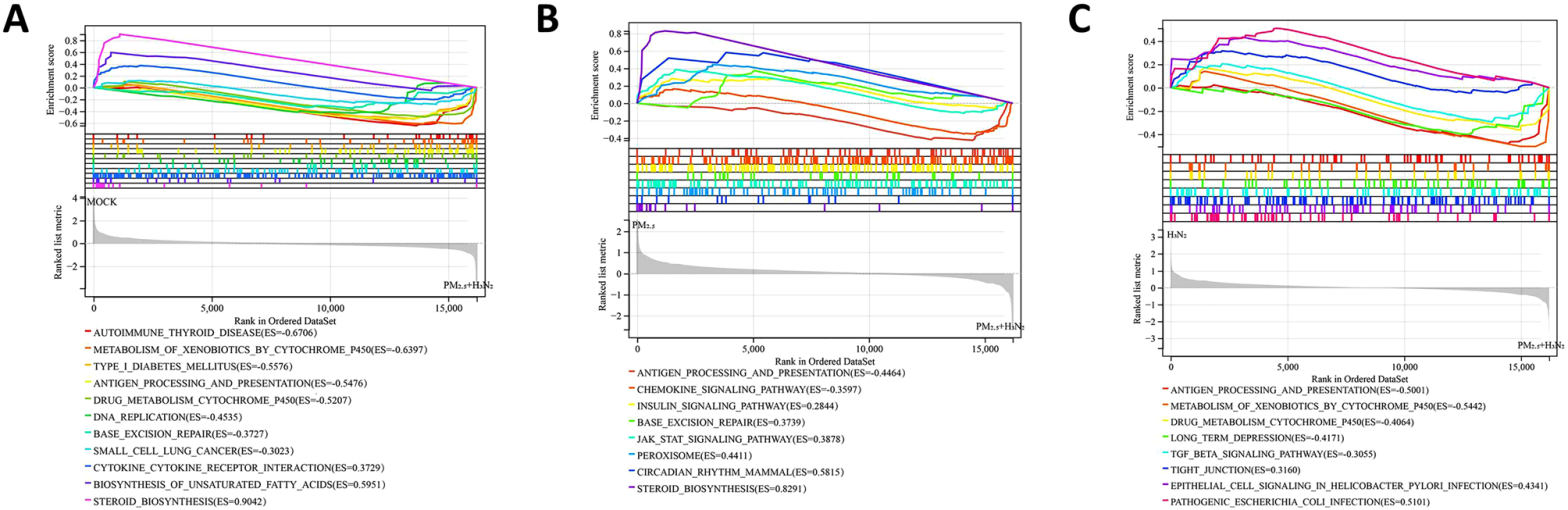
GSEA of DEGs between (**A**) Co-exposure and MOCK. (**B**) Co-exposure and PM_2.5_ exposure. (**C**) Co-exposure and H3N2 virus exposure (*P* < 0.05).

Compared with the control group, the up-regulated pathways in the co-exposure group included drug metabolism cytochrome P450, DNA replication, metabolism of xenobiotics by cytochrome P450, small cell lung cancer, base excision repair, autoimmune thyroid disease, antigen processing and presentation, and type I diabetes mellitus, the down-regulated pathways include steroid biosynthesis, biosynthesis of unsaturated fatty acids, and cytokine-cytokine receptor interaction (Fig. 8A).

Compared with PM_2.5_ group, the up-regulated pathways in the co-exposure group included the chemokine signaling pathway, antigen processing and presentation, the down-regulated pathways include base excision repair, steroid biosynthesis, circadian rhythm mammal, insulin signaling pathway, JAK-STAT signaling pathway, and peroxisome (Fig. 8B).

Compared with H3N2 group, the upregulated pathways in the co-exposure group included metabolism of xenobiotics by cytochrome P450, drug metabolism by cytochrome P450, antigen processing and presentation, and TGF -beta signaling pathway, the down-regulated pathways include epithelial cell signaling in helicobacter pylori infection, tight junction, and pathogenic Escherichia coli infection (Fig. 8C).

### Verification of DEGs using RT-PCR

The expression of some representative DEGs identified by transcriptomic methods within different groups was verified using RT-PCR. It was shown that mRNA levels of some DEGs including *CYP1A1, CYPIB1* and *Aldehyde Dehydrogenase 3 Family Member A1 (ALDH3A1)* in the co-exposure group were significantly higher than those in the control group and H3N2 group. Such results provide clues for exploring the mode of action of PM_2.5_ in combined exposures.

**Figure 9:**
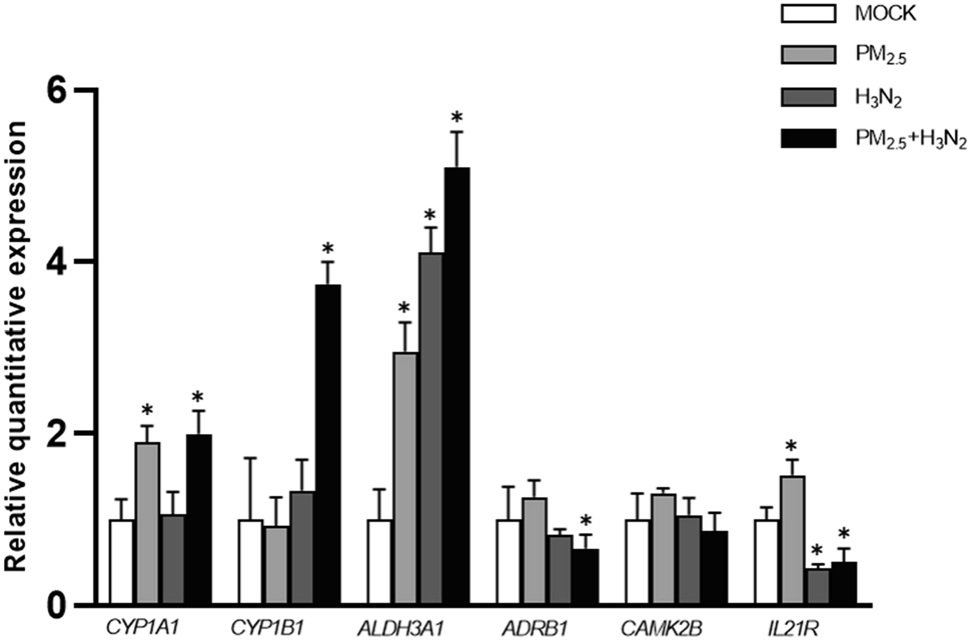
mRNA expression analysis. Detection of mRNA expression in BEAS-2B cells by RT-PCR (n = 3, *P < 0.05, compared to MOCK).

The mRNA level of *Interleukin 21 Receptor (IL21R)* was significantly higher in the PM_2.5_ exposure group and significantly lower in the H3N2 and co-exposure groups. Furthermore, the mRNA level of *ADRB1* was significantly lower in the co-exposure group, but not in the exposure alone group (*P* < 0.05), and *CAMK2B* was similar, with a tendency of lower expression in the co-exposure group. (Fig. 9). These findings were in support of the results from RNA-seq, as depicted above.

## Discussion

Even though PM_2.5_ air pollution has been significantly reduced in recent years, it remains a major threat to public health. A previous study has reported that PM_2.5_ has an impact on the infection and severity of infectious diseases^29^. In this study, we examined the joint effect of PM_2.5_ and H3N2 on gene transcription in human bronchial epithelial cells using RNA-seq. The results suggest that pleiotropic genes and pathways are involved in the promotive effect of PM_2.5_ on H3N2 infection of human bronchial epithelial cells.

To explore the effect of PM_2.5_ exposure on H3N2 infection of BEAS-2B cells and the possible underlying mechanisms, we first examined the differential effect of the co-exposure group and PM_2.5_ group on gene expression, 22 DEGs are found up-regulated and 30 DEGs down-regulated between these two groups. Analysis of the related functions of these DEGs suggest H3N2 exposure may facilitate viral protein interaction with cytokine and cytokine receptor, and epithelial cell signaling in Helicobacter pylori infection in the co-exposure group are significantly enriched pathways. Whether H3N2 exposure modulates the effect of PM_2.5_ on BEAS-2B cells through signaling molecules and interaction of environmental information processing and bacterial infectious diseases is currently under the assumption.

Previous studies have reported the interaction between viral and bacterial infections. TLR4 is proposed as unable to recognize viruses, but RSV infection has been shown to upregulate TLR4 expression, which increases inflammatory signaling and makes the respiratory system more sensitive to LPS, the major surface membrane component present in almost all Gram-negative bacteria and also a common biological component absorbed on PM_2.5_.^30^ Influenza infection followed by secondary bacterial pneumonia is associated with significant mortality and mortality. The susceptibility to bacterial infection may be increased by viral pathogen-associated molecular patterns (PAMP) desensitization to TLRs. Desensitization results in reduced chemokine production and NF-κB activation^31^. The hygiene hypothesis for asthma pathogenesis is also based on the state of tolerance after repeated PAMP exposure, early exposure to viral PAMP may lessen the risk of developing high inflammation later in life^32^. In another of our previous experiments^28^, the effects of PM_2.5_ exposure on influenza virus (H3N2) infection and downstream regulation of inflammatory and antiviral immune responses were investigated, also using the human bronchial epithelial cell line BEAS-2B. The results showed that exposure to PM_2.5_ alone increased the production of pro-inflammatory cytokines, including interleukin-6 (IL-6) and IL-8, but decreased the production of the antiviral cytokine interferon-β (IFN-β) in BEAS-2B cells. In contrast, exposure to H3N2 alone increased the production of IL-6, IL-8, and IFN-β. In this study, PM_2.5_ exposure significantly increased *IL21R* expression, whereas *IL21R* expression was decreased in both groups with H3N2 exposure. A recent study has shown that IL-21R signaling suppresses IL-17^+^ gamma delta T cell responses and production of IL-17 related cytokines in the lung at steady state and after influenza A virus infection^33^. These findings suggest that H3N2 may influence the effects on cells via signaling molecules and environmental information processing with bacterial infectious diseases interactions of viral proteins with cytokines and cytokine receptors, and epithelial cell signaling in combined exposures.

Meanwhile, we identified 32 up-regulated and 15 down-regulated DEGs between the co-exposure group and the H3N2 group. The metabolism of xenobiotics by cytochrome P450, complement and coagulation cascades, and chemical carcinogenesis are significantly enriched pathways associated with these DEGs. Whether PM_2.5_ exposure modulates the response of BEAS-2B cells to H3N2 through the pathways regulated by cytochrome P450 metabolism, respiratory immune system, or cancer-related diseases, needs to be clarified in the future.

Moreover, the expression of some DEGs in BEAS-2B cells exposed to PM_2.5_ and H3N2 are examined using RT-PCR. Similar to the results from RNA-seq, mRNA levels of *CYP1A1* and *ALDH3A1* in the co-exposure group, those of *CYP1B1* and *ALDH3A1* H3N2 group are significantly higher than those in the control group, respectively. KEGG indicates that the pathway related to *ALDH3A1* is associated with the metabolism of xenobiotics by cytochrome P450 and chemical carcinogenesis. Additionally, differential expression of *CYP1A1* and *CYP1B1* is detected between the co-exposure group and control group or H3N2 group, respectively. The *CYP1* (cytochrome P450 1) family has two significant subtypes, *CYP1A1* and *CYP1B1*, which are abundant in lung tissues. *CYP1A1* and *CYP1B1* participate in the metabolism of lung polycyclic aromatic hydrocarbons (PAHs) as PAHs-sensitive genes and can be activated by PAHs in the lung through the aryl hydrocarbon receptor (AhR)^34–36^. *CYP1A1* is most abundant in alveolar type II cells and endothelial cells^37^, while *CYP1B1* is most abundant in airway epithelial cells^35^. Interestingly, PAH content contributes to PM_2.5_ toxicity, and recent research has shown that PAHs in PM are a powerful mediator of health effects^38–41^. Mice exposed to high PM concentrations in Fresno, California were found to increase *CYP1A1* expression in pulmonary tissues, including pulmonary blood vessels, parenchyma tissue, and airways^42^. Another study found that CYP1B1, as an enzyme with a unique tumor-specific expression pattern, can bioactivate a wide range of carcinogenic compounds. Inflammatory cytokines such as tumor necrosis factor-α (TNF-α) and AhR ligands co-regulate *CYP1B1* expression and change the metabolism of exogenous carcinogens, increase the biological activity of promutagens, such as benzo[a]pyrene (BaP) in epithelial cells^43^. Recent studies have shown that CYP450 is able to influence macrophage inflammatory signaling through the PPARa axis, which may explain how PM_2.5_ affects the role of H3N2 viruses in co-exposure^44^. These findings imply that the action pattern of PM_2.5_ may be closely related to the metabolism of cytochrome P450 in the context of co-exposure to H3N2 and PM_2.5_.

Notably, this study discovered that some differential genes and pathways were identified and enriched only in the common exposure group, KEGG pathways such as calcium signaling pathway, long-term potentiation, and related DEGs including *ADRA1B*, *ADRB1*, *CAMK2B*, and so on, and the results of RT-PCR assay confirmed the DEGs differences among them. *ADRA1B* and *ADRB1* belong to the G protein-coupled receptor adrenergic receptor group and GO annotations for this gene include G protein-coupled receptor activity α1 adrenergic receptor activity, and β adrenergic receptor activity. Calmodulin-dependent kinases (CaMK) are a family of serine/threonine kinases that mediate many of the second messenger effects of Ca^2+^. Recent studies have demonstrated that CAMK2B expression is modified in neuropsychiatric illnesses and potentially affects synaptic plasticity^45^. These findings suggest that co-exposure to PM_2.5_ and H3N2 may be able to affect the calcium-sensing receptor on the cell surface by modulating Ca^2+^, which, as a typical nutrient-sensing G-protein-coupled receptor, is activated and modulated by a wide range of endogenous or exogenous substances (e.g., cations, amino acids, polyamines, aminoglycoside antibiotics, etc.), resulting in an "additional" effect^46^. Its specific mechanisms will be investigated further.

In conclusion, PM and H3N2 still pose serious risks to public health, but the mechanisms of their combined action remain largely unknown. This study profiles the transcriptome of human bronchial epithelial cells exposed to PM_2.5_ influenza virus (H3N2) by RNA-Seq. The results indicate that PM_2.5_ exposure disrupts the expression of CYP-coding genes, further altering the body's metabolism of exogenous harmful substances, and leading to the intensification of H3N2 invasion of BEAS-2B cells. Meanwhile, by working as a PAMP, H3N2 exposure might influence the immune system's response to PM_2.5_ that contains bacterial pathogens or LPS. We also found that the combined effects of PM_2.5_ and H3N2 are not simply additive and that the combined exposure of the two may have an "additional" effect by modulating Ca^2+^ to affect G protein-coupled receptors on the cell surface. The exploration of the joint effects of PM_2.5_ and H3N2 may provide insights into the pathophysiological basis of the interaction between PM and influenza virus, as well as for developing efficient strategies to prevent the adverse respiratory effects caused by PM_2.5_ and H3N2 viruses.

## Materials and methods

### PM_2.5_ collection and suspension preparation

Quartz sampling filters were used to collect PM_2.5_ in January and March 2021 using a TischTE-6070 high-flow particle sampler (Tisch Environmental, USA) with a flow rate of 1.13 m^3^/min. After sample collection, the quartz sampling filter was submerged in a Petri dish 10 cm in diameter filled with ultrapure water and sonicated three times for five minutes each. The suspension was filtered through six layers of gauze and then underwent lyophilization to collect PM_2.5_ powder using a vacuum freeze drier (Christ, Germany) for 24 h. PM_2.5_ was thoroughly suspended in phosphate-buffered saline (PBS, Solarbio Life Sciences, China) solution at a final concentration of 1 mg/mL. The suspension was aliquoted and stored in a -80 freezer. Before use, PM_2.5_ suspension was vortexed for homogenization.

### Analysis of PM_2.5_ component

1.0 mg of PM_2.5_ was digested in the mixture of 65% HNO_3_ and 30% H_2_O_2_ (3:2). The digestion condition in the microwave was 160 °C for 20 min and 140 °C for another 2 h. Metals in PM_2.5_ including magnesium (Mg), aluminum (Al), calcium (Ca), manganese (Mn), barium (Ba), copper (Cu), zinc (Zn), strontium (Sr), tin (Sn), lead (Pb), etc. were measured with inductively coupled plasma-mass spectrometry (ICP-MS, NCS testing technology, China).

### Cell culture and treatment

BEAS-2B cells (ATCC, Rockville, Maryland, USA) were used as the in vitro cell model and cultured in a cell incubator at 37 °C with 5% CO_2_ in Dulbecco’s Modified Eagle Medium (DMEM) with 10% fetal bovine serum (FBS), 100 U/mL penicillin, and 100 μg/mL streptomycin. Cells were grown in 12-well plates at a density of 10^5^/mL. Upon 90% confluence, the cells were subjected to the following treatments: serum-free medium (control group, mock), PM_2.5_ (12.5 μg/mL), H3N2 (MOI = 1), or co-exposure of PM_2.5_ (12.5 μg/mL) and H3N2 (MOI = 1).

### Cytotoxicity assay

Cell counting kit-8 (CCK-8, Shanghai Omo Biotech) was used to evaluate the cytotoxicity of PM_2.5_ and H3N2, respectively. In detail, 100 μL of 10^5^ cells /mL BEAS-2B cells were seeded into each well of a 96-wells plate and cultured at 37 °C in an incubator. Upon 90% confluence, the cells were incubated with different concentrations of PM_2.5_ (0, 6.25, 12.5, 25, 50, 100 μg/mL), H3N2 (1 MOI), and co-exposure of PM_2.5_ (12.5 μg/mL) and H3N2 (MOI = 1) for 24 h, respectively. The optical density at 450 nm was measured with an enzyme-labeled instrument (PerkinElmer, USA), and the cytotoxicity was calculated according to the manufacturer’s instructions.

### Immunoperoxidase monolayer cell assay (IPMA)

IPMA with 3-Amino-9-Ethylcarbazole (AEC) peroxidase substrate as the chromogenic solution was used to visualize H3N2-infected BEAS-2B cells. The 12-well plate was cleaned once with PBS before addition of 5% skimmed milk to each well, and the wells were then blocked in a thermostat at 37 °C for 1 h. After blocking, monoclonal antibodies against hemagglutinin of H3N2 were incubated at 37 °C for 1 h, and then washed five times with PBS containing 0.05% Tween-20 (PBST). Goat anti-mouse IgG-horseradish peroxidase (HRP) antibody (Beyotime, China) was added and incubated for another 1 h at 37 °C. AEC chromogenic solution (Affinity Biosciences, USA) was applied to identify the infected cells. The number of infected cells was counted under a microscope (Leica, Germany) which showing a brownish-red color after treatment with AEC chromogenic solution were observed and counted. The average number of H3N2-infected cells per microscopic field was determined according to a protocol for a systematic randomization procedure^47^, and 8 images were recorded for each experimental group, from 3 independent experiments.

### RNA isolation and library preparation

Total RNA was extracted using the miRNA Isolation Kit (mirVana™, Ambion-1561) according to the manufacturer's instructions. The NanoDrop 2000 spectrophotometer was used to assess the purity and quantity of RNA (Thermo Scientific, USA). The Agilent 2100 Bioanalyzer was used to evaluate the integrity of the RNA (Agilent Technologies, Santa Clara, CA, USA). The mRNA libraries were then created using the TruSeq Stranded mRNA LT Sample Prep Kit (Illumina, San Diego, CA, USA) for carrying out the transcriptome sequencing and analysis (OE Biotech Co., Ltd., Shanghai, China).

### Quality control and RNA sequencing

PCA was used to analyze gene expression data and used to measure the distance between the samples in order to identify sample similarities. Pearson correlation analysis was used to calculate the correlation coefficients between samples, and the correlation between samples represents the degree of similarity between samples, and the similarity of samples from various treatments or tissues in terms of expression levels. The correlation of biological duplicates can be used to not only examine the reproducibility of biological experimental manipulations but also to evaluate the dependability of differentially expressed genes and to aid in the screening of aberrant samples.

On the Illumina HiSeq X Ten platform, the mRNA libraries were sequenced, and 150 bp paired-end reads were generated. The HiSeq X Ten System was specially created for population-scale whole-genome sequencing. Each HiSeq X System is capable of 30-fold or greater coverage of human genome sequencing. Trimmomatic^48^ was utilized for the processing of raw data (raw reads). To get the clean reads, the low-quality reads and reads containing ploy-N were eliminated. The human genome (GRCh38) was then mapped using HISAT2^49^ using the clean reads. FPKM^50^ of each gene was calculated using Cufflinks^51^, and the read counts of each gene were obtained by HTSeqcount^52^. Differential expression analysis was performed using the DESeq (2012) R package.

### Differential expression gene (DEG) analysis

Only genes with count mean values larger than 2 were kept for the subsequent study after the genes had initially been filtered based on the count's mean value. The DESeq software was used to normalize the counts of each sample gene (the BaseMean value was used to estimate the expression), the difference fold was calculated and the NB (negative binomial distribution test) was used to test the significance of differences. Finally, the differential protein-coding genes were screened based on the fold and significance test results. For screening differences, the default parameters were *P* < 0.05 and |Log_2_foldchange|> 1. The expression pattern of genes in different groups and samples was displayed using hierarchical cluster analysis of DEGs.

### GO and KEGG enrichment analysis

R software was used to analyze the DEGs' Gene Ontology (GO) enrichment and Kyoto Encyclopedia of Genes and Genomes (KEGG) pathway enrichment based on the hypergeometric distribution. GO enrichment and KEGG^53^ pathway enrichment analysis of DEGs were performed respectively using R based on the hypergeometric distribution. Biological process, cellular composition, and molecular function, the three categories of GO functional annotations, were all covered. The pathway diagram was derived from the KEGG database.

### Gene set enrichment analysis

For Gene set enrichment analysis (GSEA), the GSEA software (version 3.0) was used to divide the samples into two groups according to the presence or absence of PM_2.5_ exposure and download the samples from Molecular Signatures Database (http://www.gsea-msigdb.org/gsea/downloads.jsp)^54^, the c2.cp.kegg.v7.4.symbols.gmt subset was downloaded to evaluate relevant pathways and molecular mechanisms based on gene expression profiles and phenotypic groupings, setting a minimum gene set of 5 and a maximum gene set of 5000. One thousand resamples with *P* < 0.05 was considered statistically significant.

### Real-time quantitative reverse transcription polymerase chain reaction (RT-PCR)

Each RT reaction contained 10 μL of 5 × *TransScript* All-in-one SuperMix for qPCR, 2 μL of 0.5 μg RNA, and 0.5 μL of gDNA Remover. In a GeneAmp^®^ PCR System 9700 (Applied Biosystems, USA), reactions were carried out for 15 min at 42 °C and 5 s at 85 °C. After being diluted 10 times in nuclease-free water, the 10 μL RT reaction mix was kept at -20 °C.

RT-PCR was carried out using the LightCycler^®^ 480 II Real-time PCR Instrument (Roche, Switzerland) and a 10 μL PCR reaction mixture that contained 1 μL of cDNA, 5 μL of 2 × *PerfectStart™* Green qPCR SuperMix, 0.2 μL of forward primer, 0.2 μL of reverse primer, and 3.6 μL of nuclease-free water. In a 384-well optical plate (Roche, Switzerland), reactions were incubated for 30 s at 94 °C, then underwent 45 cycles of 5 s at 94 °C and 30 s at 60 °C. Each sample was run in triplicate for analysis. Melting curve analysis was performed following the PCR cycles to confirm the precise generation of the desired PCR product. mRNA sequences obtained from the NCBI database served as the basis for the design of primer sequences. The primer sequences are shown in Table 4. The expression levels of mRNAs were normalized to ACTB (β-actin) and calculated using the 2-ΔΔCt method.

**Table 4:** RT-PCR primers.

Gene	Forward primer(5′ → 3′)	Reverse primer(5′ → 3′)
*ACTB*	CATTCCAAATATGAGATGCGTT	TACACGAAAGCAATGCTATCAC
*ADRB1*	GCACAGCAGATAGAAAGACTT	ATTGACAGAGTCACATGTCAC
*ALDH3A1*	GCAACGACAAGGTGATTAAGAA	GGTGATGTGGACGATGAC
*CAMK2B*	GTTTGAGCCTGAAGCACTG	GATCGGCTTGCTGTTCTT
*CYP1A1*	CCTCCAAGATCCCTACACT	CCCTGATTACCCAGAATACCA
*CYP1B1*	TCGAGTGGGAGTTAAAGCTTC	TAGGGCAAGACGTCAACAG
*IL21R*	TCGGGTTGGAAGTCAGCA	GCATTCTCTCAGCTACCTC

### Statistical analysis

SPSS 22.0 (IBM, USA) and GraphPad Prism8 (GraphPad Software, USA) statistical software was used to process the experimental data, analyze the variables, and create graphs. Data with a normal distribution were expressed as mean and standard deviation (${\overline{\text{X}}}$ ± SD). ANOVA was applied to compare the cell viability of each group, and the Games-Howell method was used to compare the groups pairwise. The t-test of two independent samples was used to compare the numbers of virus-infected cells between the combined group and H3N2 virus group in the IPMA assay, with α = 0.05.

## Data Availability

The gene expression datasets generated and/or analysed during the current study are available in the GEO repository, https://www.ncbi.nlm.nih.gov/geo/query/acc.cgi?acc=GSE244959 (Token: ijorammgfpsjjkh).
